# Nutritional Knowledge and Self-Reported Nutritional Practice against Malnutrition among Physicians in Jeddah, Saudi Arabia

**DOI:** 10.3390/healthcare7040149

**Published:** 2019-11-19

**Authors:** Areej Ali Alkhaldy

**Affiliations:** Clinical Nutrition Department, Faculty of Applied Medical Sciences, King Abdulaziz University, P.O. Box 80215, Jeddah 21589, Saudi Arabia; aalkhaldy@kau.edu.sa; Tel.: +966-12-6400000

**Keywords:** nutrition, knowledge, physicians, malnutrition

## Abstract

The new era of healthcare emphasizes the integration of nutritional care into healthcare management to improve patient outcomes. Previous studies indicated that nutritional knowledge among physicians is insufficient. Thus far, only a limited number of studies have assessed nutritional knowledge among Saudi physicians, without providing data regarding their views toward practice against malnutrition. Therefore, the aim of the present cross-sectional study was to address this knowledge gap among Saudi physicians in the hospitals of Jeddah, Saudi Arabia. A total of 117 physicians were recruited using a questionnaire to collect physician characteristics, nutritional knowledge, and knowledge and practice related to malnutrition. The mean nutritional knowledge scores were low (50%; SD: 24%). Saudi physicians scored high in questions related to the medical field; however, their knowledge related to nutrition topics was poor. The majority of Saudi physicians agreed that the nutritional management of malnourished patients was difficult at screening (79%), assessment (78%), and treatment (78%) stages. The self-assessed knowledge and interest of physicians toward malnutrition was modest, and they found the malnutrition management to be only moderately relevant to their work. Nutritional knowledge and practice against malnutrition among Saudi physicians is insufficient. Saudi physicians require proper education and training in nutrition.

## 1. Introduction

Nutrition is an important factor in the maintenance and the promotion of good health, the prevention of malnutrition and disease, and the treatment of chronic disease throughout life [1]. Collaboration of an interdisciplinary healthcare team is required, especially in the hospital setting, to ensure high-quality nutritional care [2,3]. The new era of quality care requires the involvement of a dietitian in the healthcare team (i.e., nurses, pharmacists, and physicians) to perform a nutritional assessment/diagnosis and develop evidence-based interventions [3]. Moreover, the healthcare team must have a fundamental level of nutritional knowledge to address critical issues in a more deliberate and holistic approach [4]. For example, nurses may perform the initial nutrition screening and develop a plan to facilitate patient compliance, pharmacists evaluate drug-nutrient interactions, and physicians oversee the overall care plan and documentation to support reimbursement for services [3]. Therefore, current research supports the inclusion of nutrition courses in the curriculum of health programs as well as for continuing health education [5,6,7,8].

As physicians are responsible for the overall care plan and documentation, inadequate nutritional knowledge may affect the medical treatment plan and reduce the quality of patient outcomes. Therefore, physicians are expected to have adequate nutritional knowledge, which needs to be applied in situations such as giving general nutritional advice in response to patient questions during hospital rounds or in the outpatient clinic setting as well as in practical situations such as the management of malnourished patients. Numerous studies have indicated that nutritional knowledge among physicians is insufficient relative to current recommendations [7,9,10,11]. Research worldwide (i.e., Western studies in the USA [9], Canada [5], and Europe [6,7]; Asian studies such as in Taiwan [12], Iran [13], Turkey [14]; and the Gulf countries, such as in Kuwait [15], Saudi Arabia [16], and Qatar [17]) have reported nutritional knowledge among physicians as being poor and insufficient. In addition, several studies have evaluated the attitudes of physicians along with their self-perceived proficiency. They found that confidence and knowledge among physicians to effectively provide nutritional advice in their daily practice against malnutrition is lacking [7,9,11].

Malnutrition is a highly prevalent condition in the hospital setting, particularly among the elderly and children [18,19]. It is associated with increased morbidity, mortality, prolonged hospital stays, clinical complications, and increased healthcare costs. There are no available statistics of malnutrition in Saudi Arabia; however, a study by Alzahrani et al. in Jeddah reported a high prevalence of malnutrition among geriatric outpatients (33% of total *n* = 152) and inpatients (76% of total *n* = 248) [20,21]. Therefore, including nutrition care as an early identification of malnutrition is considered important in planning for nutritional interventions that could help to reduce or prevent malnutrition-associated poor outcomes. In Saudi Arabia, there is a paucity of data regarding nutritional knowledge and nutritional practice against malnutrition among physicians. Therefore, the aim of this study was to: (1) assess the nutritional knowledge of physicians, and (2) evaluate the self-reported nutritional practice against malnutrition in the hospitals of Jeddah, Saudi Arabia.

## 2. Materials and Methods

### 2.1. Study Sample and Recruitment

This cross-sectional study was conducted between January and April 2019. A total of 117 physicians were recruited and requested to complete an online questionnaire. The sample size was calculated using the online Epi Info sample size calculator supported by the Division of Health Informatics and Surveillance, and the Center for Surveillance, Epidemiology and Laboratory services [22]. The data were obtained from the Saudi General Authority for Statistics (2017), including an estimated total of 4425 physicians in Jeddah city. The effective sample for this study was *n*  = 103 with an 80% confidence interval, margin error of 5%, and design effect of 1. The questionnaire was distributed via social media platforms, including Twitter and WhatsApp, and circulated through email by three Saudi physicians based in Jeddah. The study was approved by the Unit of the Biomedical Ethics Research Committee at King Abdulaziz University in Jeddah, Saudi Arabia (Reference No. 543-17). All participants provided electronic informed consent, and a statement of anonymity and confidentiality was included.

### 2.2. Study Measures

The study questionnaire was adapted from those published by Temple [5] and Alnumair [23] to assess the theoretical knowledge (general nutritional knowledge) and by Mowe et al. [7] to assess the practical knowledge (practice against malnutrition) with some modifications. For example, two questions (typical daily salt for Canadians and common nutrient deficiency in alcoholics) from the Temple questionnaire were removed, as these questions were not relevant to Saudi culture. They were replaced by two questions from a previous study performed in Saudi Arabia by Anumair [23], which also used a modified Temple questionnaire. These two questions were regarding the intentions of dietary recommendations and the reason for successful short-term weight loss. Two additional questions were also added during the pilot test phase of the questionnaire based on suggestions from a panel consisting of two Ph.D. holders in Clinical Nutrition, two dietitians, and two physicians. One question was about the recommended dietary allowances for calcium in adults aged 19–50 years, and the other was about the food that has the lowest glycemic index. For pilot testing, the questionnaire was shared with two Ph.D. holders in Clinical Nutrition, two dietitians, and two physicians. The questionnaire was subsequently revised based on their responses and feedback. Briefly, changes made to the pilot questionnaire included the addition of questions and the revision of response options. As a result of these modifications, the final questionnaire was composed of three sections with a total of 33 questions and required approximately ten minutes to complete.

Section one was used to collect the characteristics of the physicians including profession, specialization, age, sex, nationality, type of work facility, years of employment, educational level, and country of education.

The second section was used to measure the nutritional knowledge of the physicians (non-practical knowledge) using a questionnaire consisting of 18 multiple-choice questions, each with four possible answers, of which only one was correct. Each correct answer was assigned one point, while an incorrect answer scored zero points; consequently, the level of nutritional knowledge was assessed as a mean percentage for the correctly answered questions. If more than two-thirds (~66%) of the physicians correctly answered any of the questions, the score of the question was considered as high.

In the third section, we assessed practical knowledge concerning the three main areas of nutritional practice (i.e., screening—identify patients at nutritional risk; assessment—techniques for identifying malnourished patients; and treatment—organize a nutrition program by providing initial advice, recognizing the need for referral for more specialized nutrition therapy and coordinating overall treatment plans) in relation to the guidelines established by the European Society of Parenteral and Enteral Nutrition [24]. Physicians responding to the good clinical nutritional practice were required to select answers from a four-point Likert scale: 1 = disagree; 2 = somewhat disagree; 3 = somewhat agree; and 4 = agree. In addition, in this section, we asked the physicians to assess their knowledge, interest, and relevance of nutrition therapy on a scale from 1 (lowest) to 10 (highest). These scores were categorized as either poor (1–3 points), average (4–7 points), or good (8–10 points).

### 2.3. Statistical Analysis

Data were analyzed using the SPSS software (Version 23.0; IBM Corp., Armonk, NY, USA). Data were described using frequency statistics. Associations between the total nutrition score and the main study variables (profession, specialization, age, sex, type of work facility, years of employment, educational level) were tested using analysis of variance. Associations between these study variables and malnutrition screening, assessment, and treatment were examined using Fisher’s exact test. A *p* < 0.05 denoted statistical significance. A Bonferroni correction was applied for multiple comparisons.

## 3. Results

### 3.1. Sample Characteristics

Table 1 shows the characteristics of the physicians. The sex distribution was approximately equal (*n* = 56; 48% males and *n* = 61; 52% females). The most frequently reported characteristics among physicians were as follows: Saudi (*n* = 99; 85%), working in a governmental hospital (*n* = 99; 85%), educated in Saudi Arabia (*n* = 82; 74%) to Bachelor’s degree level (*n* = 69; 59%), aged ≤30 years (*n* = 42; 50%), working in a resident position (*n* = 49; 43%), with <2 years of experience (*n* = 47; 40%), and within the area of internal medicine (*n* = 42; 38%).

### 3.2. Nutritional Knowledge Questionnaire

Table 2 shows the number and the percentage of correct answers for each question (*n* = 18). The mean percentage of correct answers was 50% (standard deviation [SD]: 24%). Most physicians (62%) scored ≤50%; 38% of the physicians scored <75%, and only 2% scored >75%.

Of the participants, 96% were able to correctly identify folate as “the nutrient strongly associated with the prevention of neural tube defects”, which was the highest scoring question overall. The second most correctly answered question (83%) was the identification of fruit and vegetables as the “food believed to have a preventive effect on various types of cancer”. In contrast, the least correctly answered questions were those identifying pasta as the food from the available options “with the lowest glycemic index” (6% correct), followed by protein as “the nutrient for which excess may increase body calcium loss” (18% correct), and lastly that 20–35% is the “percentage of the daily total energy that should come from fat” (21% correct).

Table 3 shows that the total nutrition score for physicians increased significantly with age (*p* = 0.01) and years of employment (*p* = 0.045) and was highest among physicians with a Master’s degree (*p* < 0.001).

### 3.3. Self-Reported Nutritional Knowledge and Practice against Malnutrition

As illustrated in Table 4, approximately 80% of the physicians agreed (34%) or somewhat agreed (45%) that they have difficulty in identifying patients at nutritional risk (screening), without significant differences observed among the main study variables. Most participants (78%) agreed or somewhat agreed that they lacked techniques for identifying malnourished patients (assessment). The educational level was a significant variable in the level of agreement with this statement, with physicians holding a Master’s degree being less likely to report the lack of techniques. Similarly, most of the participants (78%) agreed that it was difficult to organize a nutritional program (treatment), without significant differences observed among the main study variables.

Table 5 presents the self-reported knowledge, interest, and relevance scores of physicians regarding the treatment of malnourished patients. The mean score for all three questions was classified as “average” (4–7 points of 10), with 6.3 (SD: 3.0) for the nutritional knowledge for the treatment of malnourished patients, 5.6 (SD: 2.8) for the interest in the treatment of malnourished patients, and 6.3 (SD: 2.9) for the relevance of being informed regarding the treatment of malnourished patients. There were no differences in the self-reported nutritional practice among the main study variables, with the exception of sex. Male physicians reported higher knowledge compared with female physicians concerning the treatment of malnutrition (mean: 7.0; SD: 3.0 vs. mean: 5.8; SD: 3.0, respectively); interest (mean: 6.1; SD: 2.7 vs. mean 5.1; SD: 2.8, respectively); and relevance (mean: 7.0; SD: 2.9 vs. mean: 5.7; SD: 2.9, respectively) of nutritional practice against malnutrition.

## 4. Discussion

In this study, we investigated the nutritional knowledge and the nutritional practice against malnutrition among Saudi physicians in the hospitals of Jeddah, Saudi Arabia. The findings of this study are relevant as physicians play an increasingly important supporting role alongside qualified dieticians in ensuring patients receive high-quality nutritional care for optimal healthcare management and prevention of disease [25,26]. Furthermore, the consistency of our findings with those of other studies from Western [5,6,7], Asian [12], and Gulf countries [15,16,17] suggests that insufficient nutritional knowledge and practice against malnutrition among physicians may be an international issue. According to Global Advances in Health and Medicine, modern healthcare should embrace the globalization of the healthcare system by providing an opportunity, not for homogenization, but for integration, convergence, and cultures collaboration to learn from our international colleagues [27]. This increasing body of evidence highlights the need to assess the medical education and training system, not just in Saudi Arabia, but worldwide, to identify factors affecting physicians’ nutritional knowledge, including the current educational system, the confidence, the knowledge, and the attitudes of the physicians toward nutrition care, and to share strategies for improvement. This step is necessary to empower the physician to be able to deliver nutrition care along with the dietitians to prevent and reduce the diet-related diseases globally. The findings and the discussion of different healthcare system are conducive to everyone’s benefit and eventually will advance the health care system internationally [27].

Overall, we found the study sample to be composed of mainly young physicians, with half of participants aged ≤30 years, 40% having <2 years of experience, and the majority (74%) educated in Saudi Arabia. This may impact the results as the lack of experience could affect their knowledge and practice.

Using the Canadian multiple-choice questionnaire devised by Temple [5], we observed that, on average, questions were answered correctly by physicians at a rate of only 50%. A similar level of correct responses was reported by Al-Zahrani and Al-Raddadi (52%) in a study conducted in Jeddah in 2009 [16], Alnumair in Riyadh (50%) in 2004 [23], and Ozcelik et al. in Turkey in 2007 (48%) [14] using the same questionnaire. However, the mean percentage for correctly answered questions in the present study was lower than that found in the survey of nutrition knowledge among physicians in Canada (63%), Qatar (64%), and Kuwait (60%). This may be attributed to differences in the studies characteristics, such as age and years of experience. In addition, some of the questions in the Temple questionnaire were changed in some of these studies, which were compared to each other; this could influence the findings. There was a range of 2–4 questions varied between the studies that were made for culture-specific reasons.

There was a high level of variation in the percentage of correct answers between individual questions with scores as low as 6% and as high as 96%, indicating inconsistency in the areas of nutritional knowledge among physicians. The results of the nutrition knowledge questions indicate that Saudi physicians are generally more knowledgeable regarding nutrition directly related to the medical field and highly publicized information. This included the role of omega-3 fatty acids in the prevention of thrombosis, hypertension, and food believed to exert a preventive effect on various types of cancer, antioxidant nutrients, and the prevention of neural tube defects (notably, questions 3, 7, 12, 14, and 15, respectively). This tendency was also reported in studies performed by Allafi in 2012 [15], Al-Numair in 2004 [23], and Temple in 1999 [5]. Relatively few physicians were able to correctly answer questions related to core nutrition topics, such as which nutrient may increase the loss of body calcium loss, which substance raises the level of blood high-density lipoprotein cholesterol, what percentage of the daily total energy should be obtained from fat, and which foods have the lowest glycemic index (questions 2, 10, 17, and 18, respectively).

Considering all the available Saudi studies conducted in the previous 15 years, including those in Jeddah in 2009 by Al-Zahrani and Al-Raddadi [16], Riyadh in 2004 by Alnumair [23], and the present study, a consistent and relatively low level of nutritional knowledge (50–52%) is evident among Saudi physicians. Furthermore, we observed that the number of correctly answered questions increased significantly with age and years of employment, which may indicate the influence of experiences and continuing medical education on improving nutritional knowledge. However, in our questionnaire, we did not include a specific question pertaining to continuing medical education or exact nutritional education. This was because the medical schools integrated the nutritional information to their updated curriculum, especially after conforming to national and international standards of the educational accreditation agencies. Although more nutritional material has been integrated into the curricula of medical schools, studies continue to report that physicians received inadequate education and training related to nutrition during their undergraduate studies and residency, respectively [9,28]. This situation may affect the interest of medical students toward the importance of nutritional care in the absence of support from their clinical house staff, who also feel that their own nutrition knowledge and counseling skills are inadequate [10,29]. In this study, a large majority of Saudi physicians agreed that they found it difficult to perform all areas of nutritional management for malnourished patients, including screening of patients on admission, assessing undernourished patients, and initiating nutritional treatment. This finding is consistent with the research performed by Mowe et al., who reported insufficient knowledge among Scandinavian doctors and nurses [7]. They and others have suggested that the inadequate nutritional knowledge often observed in hospital settings was the main barrier for performing good nutritional management for malnourished patients [7,30]. Moreover, in our study, using a scale from 1 (lowest) to 10 (highest), we observed that the self-reported knowledge of malnutrition treatment among Saudi physicians was modest (6.3) with only a moderate interest in learning more (5.6), as it was not considered highly relevant to their daily clinical practice (6.3). This may be attributed to both inadequate nutritional knowledge and the lack of proper education and training, which led to lower awareness of the importance of nutritional management in healthcare. It has been reported that medical schools do not adequately apply cognitive knowledge, different teaching methods, or the combination of theory and practical knowledge in their education system [31]. Therefore, the implementation of an integrated nutrition curriculum into the basic education using appropriate teaching methods coupled with continuing medical education after graduation is warranted. For example, some key recommendations have been published by a European Expert group to overcome the inadequate nutritional knowledge and practice in Europe [32]. They suggested a continuous education program covering general nutrition and techniques of nutritional support for all healthcare staff. In addition, education by lecturing alone might not improve self-efficacy, as it has limited opportunities for practical and clinical experiences [33]. Carson et al. reported that role modeling [34], role playing using either simulated or real patients [35], hands on practice sessions [36], and viewing videos and web-based cases and discussing them [34,36] may develop the self-efficacy of the physicians as well as their attitudes toward the nutrition care. Future work should also focus on the development of practical guidelines relevant to the medical profession, thus standards can be set that all doctors can work to as well as develop and provide resources to support the overhaul of current medical education and practice.

As for most cross-sectional studies, our study was characterized by limitations. First, its cross-sectional nature makes it difficult to establish causality. Second, we were unable to recruit all physicians practicing in Jeddah city; therefore, the small sample size may have affected the statistical power of this study and the ability to detect significant associations. This makes it difficult to generalize our findings. Furthermore, in this study, there was no certain strategy to distribute the questionnaire in terms of number of emails and physicians included in each email; therefore, we were unable to calculate the response rate, and our sample is considered as a convenience sample. Future studies need to consider this point as well as the randomization technique to be able to calculate the response rate and generalize the results. Third, our study included an online questionnaire. Therefore, we shortened the questionnaire to reduce the respondents’ burden and enhance the response rate, which could limit the information that could be obtained. Fourth, the present study was subject to bias, as the self-administered surveys do not always reflect daily clinical practice, and attitudes could be influenced by the daily workload and the physicians’ moods. However, it is generally accepted that the self-report measures of nutrition-related competences could be used as a proxy tool for actual measures of competence [37].

## 5. Conclusions

Nutritional knowledge and practice against malnutrition among Saudi physicians is insufficient. Saudi physicians require further training and continuous education regarding general nutrition. Accordingly, knowledge concerning the nutritional needs of patients should be appropriately integrated into the curricula of medical school and training programs. In addition, nutrition topics should be an essential part of continuing medical education, as most physicians have deficient nutritional knowledge, and the subject is rapidly developing. A comprehensive questionnaire assessing the nutritional knowledge and practice among physicians across all regions of Saudi Arabia is warranted. Such a study would assist in drawing key local and global recommendations that may support interdisciplinary collaboration in the new era of multidisciplinary approaches and consequently improve the quality of healthcare.

## Figures and Tables

**Table 1:** Demographic characteristics of the study sample (*n* = 117).

Variables	n ^1^	% ^2^
**Profession (*n* = 113) ^3^**		
Consultant	30	27
Fellow	15	13
Resident	49	43
Medical Intern	19	17
**Specialization (*n* = 111) ^3^**		
Internal Medicine	42	38
Pediatrics	36	32
Surgery	17	15
OB-GYN ^4^	7	6
Family Doctor	4	4
Ophthalmology	2	1
Emergency	3	3
**Age**		
≤30 years	58	50
31–40 years	37	32
≥41 years	22	19
**Years of Employment**		
≤2 years	47	40
3–5 years	24	21
6–10 years	21	18
11–20 years	12	10
21–30 years	8	7
≥31 years	5	4
**Education Level**		
Bachelor’s	69	59
Master’s	12	10
Ph.D.	36	31
**Country in which higher education was obtained (*n* = 111) ^3^**		
Saudi Arabia	82	74
Canada	4	4
USA	2	2
UK	6	5
Germany	2	2
Australia	0	0
Sweden	0	0
France	2	2
Poland	1	1
Egypt	3	3
Sudan	2	2
Combination of Saudi Arabia and another country	7	6

^1^ Number. ^2^ Percentages have been rounded and may not total to 100%. ^3^ missing data, (*n*) indicates the number of participants who completed this question if <117. ^4^ Obstetrician-gynecologist.

**Table 2:** Number and percentage of correct answers to the nutritional knowledge questions among Saudi physicians.

No.	Question (1 Point for each Question)	Responses	Correct Answer	n ^1^ (%) ^2^
1	What type of dietary fiber is helpful in lowering the level of blood cholesterol?	117	Soluble fiber	44 (38)
2	Excess of which nutrient may increase loss of body calcium?	114	Protein	20 (18)
3	A nutrient believed to help prevent thrombosis is:	116	Omega-3 fatty acids	79 (68)
4	The recommended dietary allowances for calcium in adults aged 19–50 years is:	117	1200 mg/day	35 (30)
5	The major type of fatty acids in olive oil is:	117	Monounsaturated fatty acids	43 (51)
6	Compared with unprocessed vegetable oil, hydrogenated fats contain:	116	More trans fatty acids	49 (42)
7	Which nutrient is protective against hypertension?	116	Potassium	74 (64)
8	Which vitamin is likely to be toxic if consumed in excess amounts for a long period of time?	117	Vitamin E	56 (48)
9	The most concentrated source of vitamin B12 is:	117	Meat	71 (61)
10	Which substance raises the level of blood HDL ^3^ cholesterol?	116	Alcohol	22 (19)
11	In general, dietary recommendations are intended to:	117	Maintain public health	70 (60)
12	The type of food believed to have a preventive effect on various types of cancer is:	117	Fruit and vegetables	97 (83)
13	The number of kilo-calories in one gram of fat is:	117	9	70 (60)
14	Which of the following is not an antioxidant nutrient?	117	Iron	87 (74)
15	The nutrient strongly associated with the prevention of neural tube defects is:	117	Folate	112 (96)
16	Short-term (diet) plans are usually successful at achieving weight loss because they:	117	Cause the body to lose water	64 (55)
17	What percentage of the daily total energy should come from fat?	117	20–35%	25 (21)
18	Which of the following foods have the lowest glycemic index?	117	Pasta	7 (6)
	**Average percentage of correctly answered questions**			50%

^1^ Number. ^2^ Percentages have been rounded and may not total to 100%. ^3^ High-density lipoprotein.

**Table 3:** Association between the total nutrition score and the main study variables.

Variables	Nutrition Score Mean	SD ^1^	*p*-Value ^2^
Total (*n* = 18)	8.8	2.3	
**Profession**			
Consultant	8.8	1.8	
Fellow	9.3	2.5	
Resident	7.9	2.1	0.057
Medical intern	7.9	1.7	
**Specialization**			
Internal medicine	8.3	2.2	
Pediatrics	8.4	1.7	
Surgery	8.3	2.3	
OB-GYN^3^	7.7	2.1	0.374
Family doctor	10.0	2.2	
Ophthalmology	6.0	0.0	
Emergency	10.0	2.0	
**Age**			
≤30 years	7.9	1.9	
31–40 years	8.3	1.9	
≥41 years	9.4	2.1	0.010
**Sex**			
Male	8.2	2.3	
Female	8.3	1.7	0.812
**Years of employment**			
≤2 years	7.7	1.9	
3–5 years	8.3	2.1	
6–10 years	8.9	2.1	
11–20 years	9.1	2.2	0.049
21–30 years	8.3	2.1	
≥31 years	10.2	0.4	
**Education level**			
Bachelor’s	7.9	1.9	
Master’s	10.6	1.9	<0.001
Ph.D.	8.4	1.9	

**^1^** Standard deviation; **^2^** Associations between variables tested by analysis of variance; **^3^** Obstetrician-gynecologist.

**Table 4:** Association between malnutrition screening, assessment, and treatment and main study variables (*n* = 116).

Variables	I Find it Difficult to Identify Patients at Nutritional Risk (Screening)				I Lack Techniques for Identifying Malnourished Patients (Assessment)				I Find it Difficult to Organize a Nutritional Program (Treatment)			
	Agree	Somewhat Agree	Some What Disagree	Disagree	Agree	Some What Agree	Some What Disagree	Disagree	Agree	Some What Agree	Somewhat Disagree	Disagree
n ^1^ (%) ^2^	40 (34)	52 (45)	18 (16)	6 (5)	29 (25)	61 (53)	16 (14)	10 (9)	40 (34)	51 (44)	13 (11)	12 (10)
**Profession (*n* = 112)**												
Consultant	11 (12)	16 (18)	1 (1)	1 (1)	11 (12)	13 (15)	3 (3)	2 (2)	10 (11)	13 (15)	5 (6)	1 (1)
Fellow	5 (6)	5 (6)	3 (3)	2 (2)	3 (3)	7 (8)	1 (1)	4 (4)	6 (7)	5 (6)	1 (1)	3 (3)
Resident	18 (20)	18 (20)	11 (12)	2 (2)	10 (11)	27 (30)	8 (9)	4 (4)	18 (20)	19 (21)	6 (7)	6 (7)
Medical intern	6 (7)	9 (10)	3 (3)	1 (1)	4 (4)	12 (13)	3 (3)	0 (0)	6 (7)	10 (11)	1 (1)	2 (2)
Total	40 (45)	48 (54)	18 (20)	6 (7)	28 (31)	59 (66)	15 (17)	10 (11)	40 (45)	47 (53)	13 (15)	12 (13)
*p*-value ^3^	0.399				0.352				0.803			
**Age (*n* = 116)**												
≤30 years	17 (20)	27 (31)	12 (14)	2 (2)	13 (15)	32 (37)	11 (13)	2 (2)	21 (24)	24 (28)	6 (7)	7 (8)
31–40 years	16 (19)	15 (17)	3 (3)	2 (2)	9 (10)	19 (22)	3 (3)	5 (6)	10 (12)	20 (23)	4 (5)	2 (2)
≥41 years	7 (8)	10 (12)	3 (3)	2 (2)	7 (8)	10 (12)	2 (2)	3 (3)	9 (10)	7 (8)	3 (3)	3 (3)
Total	40 (46)	52 (60)	18 (21)	6 (7)	29 (34)	61 (71)	16 (19)	10 (12)	40 (46)	51 (59)	13 (15)	12 (14)
*p*-value	0.532				0.329				0.649			
**Sex (*n* = 116)**												
Male	21 (24)	22 (26)	10 (12)	2 (2)	15 (17)	28 (32)	7 (8)	5 (6)	17 (20)	24 (28)	7 (8)	7 (8)
Female	19 (22)	30 (35)	8 (9)	4 (5)	14 (16)	33 (38)	9 (10)	5 (6)	23 (27)	27 (31)	6 (7)	5 (6)
Total	40 (46)	52 (60)	18 (21)	6 (7)	29 (34)	61 (71)	16 (19)	10 (12)	40 (46)	51 (59)	13 (15)	12 (14)
*p*-value	0.615				0.947				0.771			
**Years of employment (*n* = 116)**												
≤2 years	13 (15)	24 (28)	9 (10)	1 (1)	12 (14)	28 (32)	6 (7)	1 (1)	19 (22)	19 (22)	4 (5)	5 (6)
3–5 years	9 (10)	7 (8)	4 (5)	4 (5)	3 (3)	11 (13)	5 (6)	5 (6)	8 (9)	10 (12)	3 (3)	3 (3)
6–10 years	7 (8)	11 (13)	2 (2)	0 (0)	5 (6)	12 (14)	1 (1)	2 (2)	4 (5)	13 (15)	2 (2)	1 (1)
11–20 years	5 (6)	5 (6)	2 (2)	0 (0)	4 (5)	4 (5)	3 (3)	1 (1)	3 (3)	5 (6)	2 (2)	2 (2)
21–30 years	4 (5)	3 (3)	1 (1)	0 (0)	2 (2)	4 (5)	1 (1)	1 (1)	4 (5)	3 (3)	0 (0)	1 (1)
≥31 years	2 (2)	2 (2)	0 (0)	1 (1)	3 (3)	2 (2)	0 (0)	0 (0)	2 (2)	1 (1)	2 (2)	0 (0)
Total	40 (46)	52 (60)	18 (21)	6 (7)	29 (34)	61 (71)	16 (19)	10 (12)	40 (46)	51 (59)	13 (15)	12 (14)
*p*-value	0.495				0.263				0.683			
**Educational level (*n* = 116)**												
Bachelor’s	24 (28)	29 (34)	13 (15)	3 (3)	15 (17)	40 (46)	11 (13)	3 (3)	23 (27)	31 (36)	7 (8)	8 (9)
Master’s	1 (1)	7 (8)	2 (2)	1 (1)	1 (1)	4 (5)	3 (3)	3 (3)	3 (3)	4 (5)	1 (1)	3 (3)
Ph.D.	15 (17)	16 (19)	3 (3)	2 (2)	13 (15)	17 (20)	2 (2)	4 (5)	14 (16)	16 (19)	5 (6)	1 (1)
Total	40 (46)	52 (60)	18 (21)	6 (7)	29 (34)	61 (71)	16 (19)	10 (12)	40 (46)	51 (59)	13 (15)	12 (14)
*p*-value	0.324				0.028				0.415			

^1^ Number. ^2^ Percentages have been rounded and may not total to 100%. ^3^ Fisher’s exact tests.

**Table 5:** Association between knowledge, interest, and relevance of nutritional practice and main study variables.

Variables	How Good is Your Knowledge Regarding the Treatment of Malnourished Patients? (Scale 1–10)			How Interested Are You in the Treatment of Malnourished Patients? (Scale 1–10)		How Relevant do You Find Being Informed Regarding the Treatment of Malnourished Patients? (Scale 1–10)	
	Mean	SD ^1^	Mean		SD	Mean	SD
	6.3	3.0	5.6		2.8	6.3	2.9
**Profession**							
Consultant	6.1	2.4	5.6		2.6	6.6	2.9
Fellow	6.1	3.1	6.2		2.9	6.5	2.6
Resident	6.3	3.4	5.3		3.0	5.8	3.2
Medical intern	6.8	3.1	5.5		2.5	6.8	2.8
*p*-value ^2^	0.887		0.724			0.480	
**Age**							
≤30 years	6.4	3.3	5.6		3.0	6.1	3.0
31–40 years	5.8	2.8	5.1		2.8	5.9	2.9
≥41 years	7.1	2.4	6.3		2.1	7.5	2.5
*p*-value	0.251		0.301			0.095	
**Sex**							
Male	7.0	3.0	6.1		2.7	7.0	2.9
Female	5.8	3.0	5.1		2.8	5.7	2.9
*p*-value	0.036		0.038			0.016	
**Years of employment**							
≤2 years	6.3	3.3	5.6		2.8	6.6	2.9
3–5 years	6.8	3.2	5.5		3.1	5.8	3.3
6–10 years	5.5	2.3	5.6		2.5	5.4	2.2
11–20 years	6.7	3.1	4.8		2.7	7.1	3.4
21–30 years	6.9	3.2	7.0		2.9	6.8	3.2
≥31 years	6.4	2.3	5.4		1.5	7.2	2.6
*p*-value	0.789		0.699			0.497	
**Education level**							
Bachelor’s	6.4	3.2	5.4		2.9	6.1	3.0
Master’s	6.9	3.2	6.5		2.5	6.5	3.3
Ph.D.	6.0	2.7	5.6		2.7	6.6	2.8
*p*-value	0.639		0.518			0.701	

^1^ Standard deviation. **^2^** Associations between variables tested by analysis of variance.
